# Comparative Proteomics of Chloroplasts Envelopes from Bundle Sheath and Mesophyll Chloroplasts Reveals Novel Membrane Proteins with a Possible Role in C4-Related Metabolite Fluxes and Development

**DOI:** 10.3389/fpls.2013.00065

**Published:** 2013-03-28

**Authors:** K. Manandhar-Shrestha, B. Tamot, E. P. S. Pratt, S. Saitie, A. Bräutigam, A. P. M. Weber, Susanne Hoffmann-Benning

**Affiliations:** ^1^Department of Biochemistry and Molecular Biology, Michigan State UniversityEast Lansing, MI, USA; ^2^Plant Biochemistry, Heinrich-Heine University DüsseldorfDüsseldorf, Germany

**Keywords:** C4 plant, chloroplast envelope proteins, photosynthesis, mesophyll cells, bundle sheath cells

## Abstract

As the world population grows, our need for food increases drastically. Limited amounts of arable land lead to a competition between food and fuel crops, while changes in the global climate may impact future crop yields. Thus, a second “green revolution” will need a better understanding of the processes essential for plant growth and development. One approach toward the solution of this problem is to better understand regulatory and transport processes in C4 plants. C4 plants display an up to 10-fold higher apparent CO_2_ assimilation and higher yields while maintaining high water use efficiency. This requires differential regulation of mesophyll (M) and bundle sheath (BS) chloroplast development as well as higher metabolic fluxes of photosynthetic intermediates between cells and particularly across chloroplast envelopes. While previous analyses of overall chloroplast membranes have yielded significant insight, our comparative proteomics approach using enriched BS and M chloroplast envelopes of *Zea mays* allowed us to identify 37 proteins of unknown function that have not been seen in these earlier studies. We identified 280 proteins, 84% of which are known/predicted to be present in chloroplasts. Seventy-four percent have a known or predicted membrane association. Twenty-one membrane proteins were 2–15 times more abundant in BS cells, while 36 of the proteins were more abundant in M chloroplast envelopes. These proteins could represent additional candidates of proteins essential for development or metabolite transport processes in C4 plants. RT-PCR confirmed differential expression of 13 candidate genes. Chloroplast association for seven proteins was confirmed using YFP/GFP labeling. Gene expression of four putative transporters was examined throughout the leaf and during the greening of leaves. Genes for a PIC-like protein and an ER-AP-like protein show an early transient increase in gene expression during the transition to light. In addition, *PIC* gene expression is increased in the immature part of the leaf and was lower in the fully developed parts of the leaf, suggesting a need for/incorporation of the protein during chloroplast development.

## Introduction

Changes in the world population have drastically increased our need for food and fuel. When faced with similar issues in the 1940s, the “green revolution,” led by Norman Borlaug, involved the development of high-yielding varieties of cereal grains, modernization of management techniques and irrigation systems, as well as distribution of hybridized seeds, synthetic fertilizers, and pesticides to farmers. Today, the amount of arable land is limited and often there is now a competition between food and fuel crops. In addition, changes in the global climate may impact future yields. To continue to be able to provide sufficient food and fuel, we need plants that show accelerated growth, have a higher grain, or cell wall yield/quality, and are more resistant to biotic and abiotic stressors. One adaptation of plants in response to a dry environment is C4 photosynthesis. This process allows for biomass accumulation with high nitrogen and water use efficiency (Leegood and Edwards, 1996; Sage, 2004), a trait which increases the productivity of crop plants (Matsuoka et al., 1998). During photosynthesis in the C4 plant *Zea mays*, primary CO_2_ fixation and the subsequent carbon reduction are spatially separated into mesophyll (M) and bundle sheath (BS) cells, respectively. Maize belongs to the NADP-malic enzyme type of C4 plants (Hatch, 1987). In maize mesophyll cells, CO_2_ is initially fixed *via* phosphoenolpyruvate (PEP) – carboxylase to oxaloacetate (OAA), transported into the chloroplast, and converted to malate by NADP-malic enzyme (ME). Malate moves from the surrounding mesophyll cells into BS cells and is decarboxylated in the chloroplast, yielding CO_2_, NADPH, and pyruvate. CO_2_ and NADPH enter the Calvin–Benson Cycle where CO_2_ is reduced to triose phosphates, while pyruvate is transported back to mesophyll cells, imported into chloroplasts, and converted back to the primary CO_2_ acceptor PEP by the enzyme phosphoenolpyruvate phosphate dikinase (PPDK). In addition to the enrichment of CO_2_ around Rubisco, the oxygenation reaction of the enzyme is further reduced by a limited PSII reaction and thus reduced O_2_ production in the BS chloroplast (Meierhoff and Westhoff, 1993). These processes require the shuttling of intermediates as well as reduction equivalents between cells and organelles and consequently across several membranes. As a result, chloroplasts of mesophyll and BS cells have adapted to their respective roles (Slack et al., 1969; Edwards et al., 2001; Majeran et al., 2005) and are functionally different from each other as well as from chloroplasts in C3 plants (Bräutigam et al., 2008). Despite detailed knowledge about the soluble proteins involved in and necessary for C4 photosynthesis and an increasing body of information about the chloroplast membrane proteome in both C3 and C4 plants (Bräutigam et al., 2008; Majeran et al., 2008), many aspects of the adaptation of integral and peripheral membrane proteins as well as the necessary regulatory proteins remain unknown. Here, we focus on analyzing the quantitative and qualitative differences between isolated chloroplast envelope membranes of BS and mesophyll cells, followed by localization and expression studies to further understand the possible impact of newly described envelope proteins.

Two membranes separate the chloroplast from the remainder of the plant cell: the outer and the inner envelope. Metabolite transport through the outer envelope is largely controlled through substrate-specific pore proteins (Pohlmeyer et al., 1997, 1998; Bolter et al., 1999; Goetze et al., 2006), while transport across the inner envelope is mediated by a large number of specific transporters (Weber, 2004; Weber et al., 2005; Weber and Fischer, 2007). The spatial separation between primary CO_2_ fixation and carbon reduction and the resulting necessary movement of metabolites, requires at least four transport processes. Good candidates for PEP export, triosephosphate shuttling, and oxaloacetate/malate transport have already been described: three maize homologs of the inner envelope DIT (dicarboxylate transporter), DIT1, and DIT2, likely function as 2-oxoglutarate/malate translocator and are expressed in the mesophyll envelopes and BS envelopes, respectively (Taniguchi et al., 2004; Majeran et al., 2005). The function of the third DIT homolog, also named 2-oxoglutarate/malate transporter 1 (OMT1), remains unclear (Taniguchi et al., 2004). Other putative mesophyll envelope transporters, mesophyll envelope proteins MEP 1–4, were expressed in both mesophyll and BS, whereas MEP3 in the BS (Majeran et al., 2005; Bräutigam et al., 2008). The molecular nature of others, for example the predicted pyruvate transporter, is unknown. Likewise, it is unknown whether the same or different transport proteins mediate metabolite transport across the mesophyll and the BS chloroplast envelope and whether additional transporters exist. In addition, new proteins necessary for regulating the differential development of BS and mesophyll chloroplasts may form new membrane receptors or may need to be transported into the chloroplast, thus appearing in envelope proteomes.

In this work, we compared the proteome of purified envelopes of BS and mesophyll chloroplasts to identify further components of C4 metabolite transport. We hypothesized that this enrichment step will allow us to identify differentially distributed yet less abundant and previously undescribed integral or peripheral membrane proteins as well as putative regulatory proteins imported into the chloroplast. We applied a direct quantification method, the total spectral count of proteins (number of mass spectra that map to one protein), which has been used to analyze large datasets of proteins, to compare the relative abundance of BS and mesophyll chloroplast envelope proteins (Liu et al., 2004; Zybailov et al., 2005; Lu et al., 2007; Bräutigam et al., 2008; Majeran et al., 2008). GFP labeling confirmed their localization at the chloroplast. Furthermore, we used RT-PCR to correlate the gene expression of several of the newly identified putative membrane proteins with chloroplast development and protein levels to better understand their putative function.

## Materials and Methods

### Plant material

*Zea mays* Great Lakes 4758 hybrid seeds were rinsed thoroughly to remove fungicides and shaken in water for up to 1 h to speed germination. Kernels were planted in a standard soil mixture containing equal parts of Bacto Soil (Michigan Pear Company, Houston), medium vermiculite, and perlite. Plants were grown either in complete dark for extraction of mRNA associated with development or at a 12 h day/12 h night cycle at a daytime temperature of 22°C for BS-mesophyll comparisons. For expression studies of chloroplast envelope proteins and for envelope protein preparations, plants were harvested after 6 weeks. For the light-induction experiment, plants were kept in the dark for the first 6 days of germination and then transferred to light.

### Isolation of mesophyll protoplast and BS strands

Leaves of 4–6-week-old corn plants were collected and the midrib removed. Mesophyll protoplast and BS strands were isolated following the method by Kanai and Edwards (1973) with some modifications. In short, 5 g of leaves were cut into thin slices (0.5–0.7 mm wide). Leaf slices were incubated in100 ml of digestion media (0.6 M Sorbitol; 20 mM MES (pH 5.5); 5 mM MgCl_2_, and 2% Cellulase (PhytoTechnology Laboratories, Overland Park, KS, USA). The flask was put under vacuum for 5 min, followed by incubation in a shaker (60 rpm) at 30°C for 2 h. The digestion media was discarded, 100 ml of fresh media added to the leaves, and the incubation repeated for an additional hour. Digestion media was discarded and 30 ml of 0.6 M Sorbitol was added, followed by gentle shaking for 15 min. The wash was filtered first through a tea strainer, then through 80 μm nylon filter. This process was repeated twice and the washes were centrifuged at 300 × *g* for 3 min. The supernatant was discarded and the pellet was further purified using the two phase system as described (Kanai and Edwards, 1973) to obtain pure mesophyll protoplast. The purified protoplast was stored at −80°C. BS strands collected on the 75 μm mesh was washed with Sorbitol medium [0.6 M Sorbitol, 0.05 M Tricine-KOH (pH 8.0); 5 mM MgCl_2_], mixed with a vortex for 10 s, and filtered through 75 μm nylon filter. BS strands were collected from the nylon filter and stored at −80°C.

### Preparation of chloroplast envelopes

Purified, intact chloroplast were broken in rupture buffer (10 mM Tricine/KOH pH 7.5, 1 mM PMSF, 5 mM EDTA), layered over 21% sucrose and 45% sucrose in TE, and ultra-centrifuged at 180000 × *g* for 90 min (Cline et al., 1981). The yellow band was recovered as chloroplast envelopes were collected, while the precipitate was recovered as crude chloroplast fraction. Envelope membranes were diluted with TE containing 1 mM PMSF, pelleted by centrifugation for 1 h at 25 g, resuspended in TE/PMSF, and stored at −80°C.

### Protein identification

For proteomics analysis, mesophyll and BS envelope membranes from two and three individual preparations, respectively, were dissolved in sample buffer and separated using 10% SDS–PAGE. After staining, each gel lane was cut into 10 equally sized slices. Gel slices were subjected to tryptic digest as described by Shevchenko et al. (1996) and analyzed according to Bräutigam et al. (2008). In short, peptides were loaded onto a Waters Symmetry C18 peptide trap (5 μm, 180 μm × 20 mm) at a flow rate of 4 μL/min in 2% acetonitrile/0.1% formic acid for 5 min. The peptides were separated on a Waters BEH C18 nanoAcquity column (1.7 μm, 100 μm × 100 mm) using a Waters nanoAcquity UPLC coupled to a ThermoElectron LTQ-FTICR mass spectrometer (flow rate of 300 nl/min; buffer A = 99.9% water/0.1% formic acid, buffer B = 99.9% acetonitrile/0.1% formic acid: gradient of 5% B to 40% B from 0 to 63 min, 40% B to 90% B from 63 to 71 min, and 5% B from 71 to 90 min). Survey scans were taken at a resolution of 50000 and the top 10 ions were subjected to automatic low-energy CID. The BioWorks Browser version v3.2 converted the resulting MS/MS spectra to a peak list.

### Data analysis

Scaffold^1^ was used to validate MS/MS-based peptide and protein identifications using the Peptide Prophet algorithm (Keller et al., 2002). Parameters were set at 95% confidence for protein identification requiring at least two unique peptides for each protein, and 95% confidence for all peptides counted (shown in Table S2 in Supplementary Material). Where Scaffold reported multiple proteins identified for the same peptides, each match was manually inspected and low-scoring matches were discarded. Proteins were compared to sequence databases *Zea mays*^2^. Individual matching of tryptic fragments to predicted proteins was confirmed manually. Identified proteins were imported into Microsoft Excel for further analyses.

Each sequence was compared with the Arabidopsis proteome using blastx in plprot (Altschul et al., 1997) in TAIR and the *Arabidopsis* gene identifier (AGI) of the closest homolog was recorded. Proteins were then searched against PPDB^3^. Targeting prediction and membrane-spanning regions were achieved by using the software programs TargetP (Emanuelsson et al., 2000), ChloroP (Emanuelsson et al., 1999), WoLFPSORT (Horton et al., 2006), and Octopus (Viklund and Elofsson, 2008).

### Semiquantitative analysis of protein abundance

The semiquantitative analysis of protein abundance was based on the spectral count (i.e., the number of mass spectra mapping to a given protein in a single experiment) and performed according to Bräutigam et al. (2008). In short, all proteins in the sample were separated by SDS-PAGE and identified by liquid chromatography-electrospray ionization-MS/MS without prior fractionation (“whole envelopes”). The spectral counts for each protein were summed to yield the “sum” fraction. For all five data sets, spectral counts for each protein were normalized to the total number of spectra within the experiment (“percentage of the total spectral count”; Table S3 in Supplementary Material).

### RNA isolation and RT-PCR

Total RNA was isolated from the mesophyll protoplast and BS strands using RNeasy Plant Mini Kit (Qiagen, Valencia, CA, USA) and cDNA was synthesized using SuperscriptIII Reverse Transcriptase (Invitrogen, Carlsbad, CA, USA). RNA concentration and quality were determined with a NanoDrop ND-1000 UV-Vis spectrophotometer (NanoDrop Technologies, Wilmington, DE, USA). For PCR, GoTaqGreen master mix (Promega, Madison, WI, USA) or Failsafe PCR buffers (Epicenter, Madison, WI, USA) were used. For each primer set, the optimum amount of cDNA for the PCR reaction was determined by testing a series of cDNA dilutions with a fixed number of PCR cycles.

Gene-specific PCR primers were used (Table S1 in Supplementary Material) for analyzing the abundance of the transcripts of the individual genes in mesophyll and BS samples. 18S was used as an internal control. To check for the purity of the sample, PEPC and Rubisco primers were used as markers for mesophyll and BS protoplast, respectively. PCR products were visualized by agarose gel electrophoresis and the gel image was taken using a gel documentation system (Fotodyne Inc., Hartland, WI, USA). The intensity of the bands was quantified using ImageQuant software version 5.2 (Molecular Dynamics, Sunnyvale, CA, USA). The expression levels of the individual gene in mesophyll and BS samples were compared after normalization to 18S. RT-PCR was repeated using three to five different sets of mesophyll protoplast and BS strands.

### Subcellular localization

Coding sequence for the *Arabidopsis* homologs of ERaP, 5-TM, Mep 3, UP-a, UP-d, Hyp g, and PIC were amplified using the gene-specific primers and the PCR products were cloned into pDONR207 by BP recombination reaction, sequenced, and sub-cloned into pEarleyGate103 vector by LR recombination reaction to generate the expression constructs (Earley et al., 2006). The constructs were transformed into *Agrobacterium tumefaciens* C58C1pGV2260 and the transformant cultures were used for infiltrating *Nicotiana tabacum* leaves. Expression of the GFP fusion proteins were analyzed by confocal microscopy (Carl Zeiss, USA).

### Expression studies of chloroplast envelope proteins

Roots, mesocotyl, and three sections of corn leaves were harvested and frozen in liquid nitrogen. For the leaf samples, we used a 1 cm section still inside the leaf sheath (IL), which was etiolated and is a sink tissue. Leaf sample 2 (ML), was taken from the middle of the leaf and corresponds to “part 5” of the leaf as described by Pick et al. (2011). Cells in this part of the leaf have been shown to contain developed chloroplasts and are a source tissue, however, they may still be expanding. Leaf sample 3 (LT), corresponds to “part1/2” described by Pick et al. (2011); it is a source tissue with fully developed cells. The samples were ground to a fine powder and RNA was extracted using a commercial RNA extraction kit provided by Qiagen. RNA concentrations were determined using a NanoDrop ND-1000 UV-Vis spectrophotometer (NanoDrop Technologies, Wilmington, DE, USA). For cDNA synthesis, 600 ng mRNA per sample was reverse transcribed using SuperscriptIII Reverse Transcriptase (Invitrogen, Carlsbad, CA, USA). PCR primers used for the respective genes are listed in Table S1 in Supplementary Material. Identity of the PCR products was confirmed by size and sequencing. The intensity of the bands was determined as described above and normalized to 18S. The mean and standard error was calculated from three biological replicates. Three different sets of experiments were performed: (I) BS/MS comparison, (II) distribution of the gene expression in 6-week-old plants and (III) expression changes during the development of the chloroplast. For the latter, 6-day-old dark-grown corn plants were transferred to continuous light and gene expression was studied after transition to light. RNA was extracted from primary leaves every 2 h from the start of light exposure and expression of three to six biological replicates examined as described above.

## Results and Discussion

### Identification and relative quantification of proteins associated with the envelope of BS and mesophyll chloroplasts

**Figure 1:**
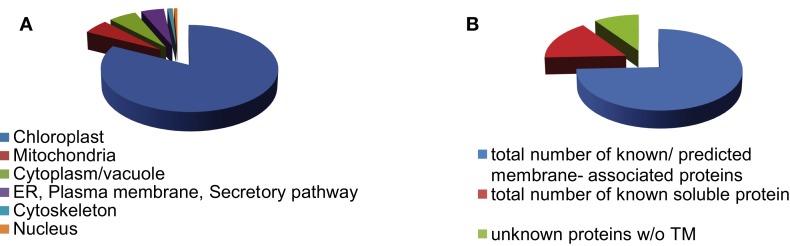
**Distribution of envelope proteins throughout different cell compartments (A) and by membrane association (B)**.

**Figure 2:**
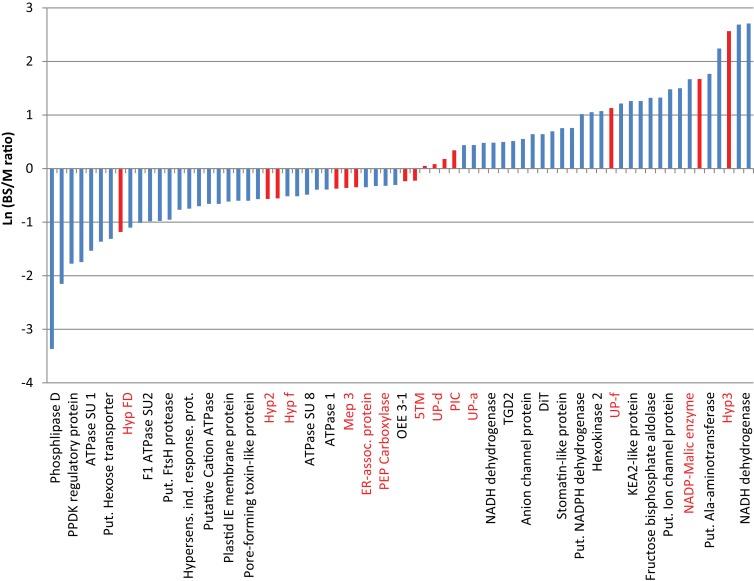
**Differences in the relative abundance of proteins based on the bundle sheath: mesophyll ratio of their spectral ion counts**. Data are based on values shown in Tables S3 and S4 in Supplementary Material. Except for known controls, only membrane-associated proteins are integrated. Due to space constrains only ca. 33% of the bars are labeled. The complete protein list and figure are shown in Table S4 in Supplementary Material. Values are the averages from two mesophyll and three BS data sets. Red bars indicate proteins used for further studies.

We identified 280 proteins in our chloroplast envelope preparations from BS and mesophyll cells (Table S2 in Supplementary Material). Of these, 84% (230 proteins) were shown to be associated with chloroplasts (WoLF PSORT^4^; Horton et al., 2006; bar.utoronto.ca; Winter et al., 2007), 5% each are localized in mitochondria or cytoplasm/vacuole, respectively (Figure 1A). About 75% of all identified proteins are known or predicted to have membrane association, while 16% are known soluble proteins and 9% are unknown proteins without transmembrane regions (Figure 1B). The majority of the soluble proteins are known chloroplast stroma proteins and were likely purified during transit across the chloroplast envelope. Despite the fact that large chloroplast proteome databases already exist, we were able to detect 37 proteins of unknown or unconfirmed function that are not present in the 15 largest databases (Peltier et al., 2002; Ferro et al., 2003; Froehlich et al., 2003; Schleiff et al., 2003; Friso et al., 2004; Kleffmann et al., 2004; Peltier et al., 2004; von Zychlinski et al., 2005; Kleffmann et al., 2006; Siddique et al., 2006; Sirpiö et al., 2007; Tyra et al., 2007; Ferro et al., 2010; Weber, 2010; Breuers et al., 2011; Fischer, 2011; Marchler-Bauer et al., 2011; Kriechbaumer et al., 2012; Lundquist et al., 2012; Majeran et al., 2012). This confirms that further fractionation of the chloroplast can lead to the discovery of more novel proteins. Determining the ratio of spectral ion counts between BS and mesophyll (M) cells revealed that 67 proteins showed possible differential abundance (as confirmed by *t*-test, *p* < 0.1; Tables S3 and S4 in Supplementary Material). A BS/M ratio of less than 0.75 indicated mesophyll association while a ratio of larger than 1.5 suggested BS localization (Figure 2). The proteins identified predominantly in the mesophyll samples contain 10 subunits of different ATPases, proteins involved in photosynthetic electron transport, as well as OEE3-1 and a FtsH proteins. This is consistent with the fact that photosystem II (PSII) is down-regulated in the BS cells of C4 plants (Meierhoff and Westhoff, 1993; Majeran and van Wijk, 2009), and as a result, proteins involved in PSII should be more abundant in mesophyll cells. This includes not only proteins directly involved in PSII electron transport but also the components of the FtsH complex, which play a direct role in the maintenance of PSII (Kato et al., 2009), and oxygen evolving enhancer proteins (OEE), which are part of the oxygen evolving system of PSII.

The proteins with higher abundance in BS cells include several enzymes of the Calvin–Benson–Bassham Cycle (Fructose-bisphosphate aldolase, Sedoheptulose-1,7-bisphosphatase; Bassham et al., 1950), sugar signaling (Hexokinase; Xiao et al., 2000), and lipid metabolism (Pheophorbide oxygenase, TGD2-like protein; Roston et al., 2011). In addition, we find a stomatin-like protein (protease), several known transporters/channels (2-oxoglutarate/malate translocator: DiT/OMT; Tic110-like protein; put. ion channel protein; KEA2-like protein, voltage dependent anion channel proteins 1a, Toc159-like protein, small drug exporter, DCT2, TGD2-like protein, DiT1, ZmPIP2-3; Jarvis and Soll, 2002; Linka and Weber, 2010; Kinoshita et al., 2011; Roston et al., 2011), as well as several proteins with a possible role in electron transport (Put. NADH-ubiquinone oxidoreductase 20 kDa SU; two distinct putative NADPH/NADH dehydrogenase proteins; Chlorophyll a–b binding protein 4; Rochaix, 2011). Proteins with a known or predicted role in C4 metabolism (NADP-dependent malic enzyme and a putative alanine aminotransferase; Pick et al., 2011) and eight proteins of unknown function were also found. Two of the proteins (putative NADH dehydrogenase LOC100282384, Hyp 3) were not found in the mesophyll envelope samples. Hyp3, however, was only identified in one of the samples, suggesting it is either in very low abundance or a cytoplasmic contamination. NADH-ubiquinone oxidoreductase/NADH dehydrogenases usually participate in mitochondrial electron transport, yet close relatives are found in chloroplasts. It is speculated that the chloroplast enzymes might use the quinone reductase function of the complex with a different reductant, perhaps ferredoxin or NADPH. This would corroborate their proposed function in the cyclic electron transport. It has been shown that in NADP-ME-type C4 plants the NDH complex is up-regulated in the BS, where it could contribute to the higher ATP requirement (Heber and Walker, 1992; Shikani, 2007; Rochaix, 2011). Similarly, the higher abundance of Calvin–Benson Cycle enzymes is not surprising, since carbon reduction through this path has been shown to occur in the BS cells (Taiz and Zeiger, 2006). Interestingly, one of the proteins with the largest differential abundance between BS and M cells is a putative alanine aminotransferase, which showed a BS/M ratio of 9.3. This corroborates a revised model for C4 photosynthesis in corn (Pick et al., 2011), which proposes that C4 metabolism branches after formation of oxaloacetate in the mesophyll cells. In this model both Asp and malate are formed and transported to the BS cell. Asp is converted by Asp aminotransferase (AspAT) to phosphoenolpyruvate and returned to the mesophyll cells; malate is decarboxylated to pyruvate which is either transported back to the mesophyll or converted to Ala *via* alanine aminotransferase (AlaAT). Ala then moves to the mesophyll where it is converted back to pyruvate by a second AlaAT (Pick et al., 2011). We have found both AspAT and a putative AlaAT in our samples, however only AlaAT shows a strong differential distribution with a higher abundance in the BS chloroplast (BS/M ratio: 9.3), while AspAT appears only marginally increased in the M chloroplast. Since AlaAT will likely be needed in both cell types, it is possible that two homologs with different expression patterns may exist or that they are in compartments other than the chloroplast and would not have been detected in our dataset.

The function of several of the predicted transporters (Tic110-like, BS/MS: 3.37; KEA-2 like, BS/MS: 3.52; Put. ion channel, BS/MS: 4.38, voltage dependent anion channel proteins 1a and 2, BS/MS: 1.58/2.00, small drug exporter BS/MS:1.62, ZmPIP2-3 BS/MS:3.75) as well as that of the hypothetical proteins (Expressed protein BS/MS: 1.64, UP-f, BS/MS: 3.09; Hyp Protein/LOC100276764 BS/MS: 4.47; UP-e, BS/MS: 5.3, UP-b BS/MS: 5.86) remains to be determined.

### Correlation of gene expression with protein abundance

**Figure 3:**
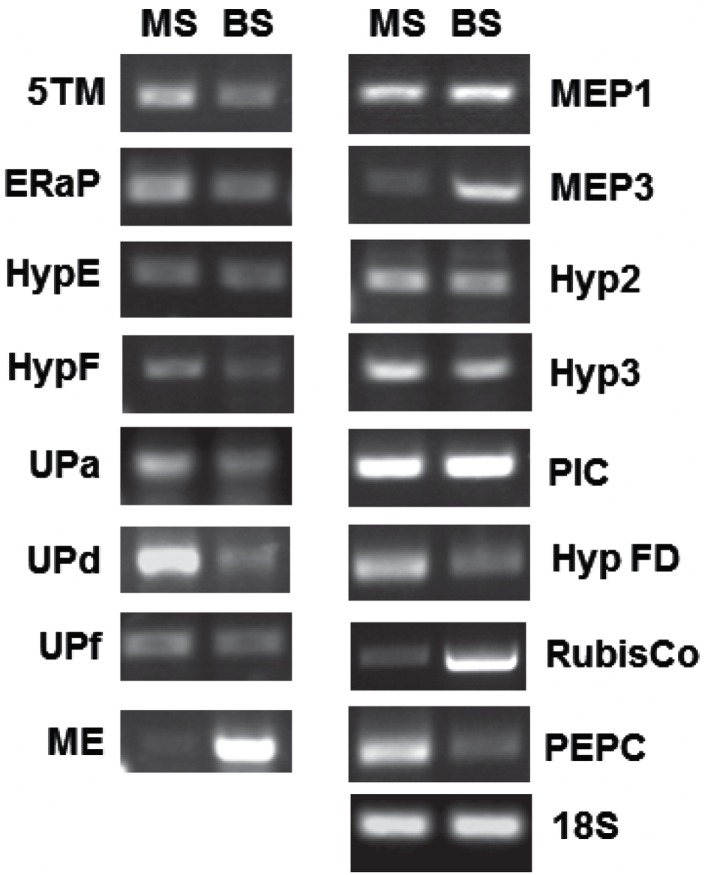
**RT-PCR showing relative abundance of transcripts for several genes encoding chloroplast envelope proteins**. Band intensities of three to five biological replicates were quantified and are displayed in Table 2. MS, mesophyll; BS, bundle sheath.

To confirm the differential abundance of several of the chloroplast envelope proteins, we picked 13 proteins of unknown function as well as two controls (M: PEPC-phosphoenolpyruvate carboxylase; BS: NADP-ME – NADP-malic enzyme; Table 1) and compared their gene expression levels in BS and MS protoplast by semiquantitative RT-PCR (Table 2; Figure 3). The proteins chosen were representatives of proteins which were more abundant in the mesophyll cells (Hyp FD, Hyp2, HypF), more abundant in the BS cells (Hyp 3, UP-f), or of equal abundance (Mep1, Mep3, ER-AP, PIC, UP-a, Up-d, 5TM, HypE). While the first and the last category may be important for M or BS-specific membrane and transport processes, the second group may be involved in chloroplast processes common to both cell types. The selected proteins were predicted to be associated with membranes or have transmembrane regions, yet their function was not well characterized (see Table 1):

**Table 1:** **List of proteins used for further study and their predicted function**.

Protein name	Accession no.	No. of TM regions (Octopus)	Predicted function
5TM	LOC100283913	8	*A.t*. homolog contains a DUF92 domain; predicted to be associated with the chloroplast inner envelope (NCBI).
ER-AP	LOC100283096	3	ER-associated protein but was also found in the chloroplast. Predicted to play a role in the formation of tubular ER in mammals and yeast (Nziengui et al., 2007).
Hyp2	LOC100285177	11	Contains a calcium-binding domain and may play a role in calcium modulation or signaling. Its pfam01699 domain suggests a possible role as a Sodium-Calcium exchange protein (NCBI).
Hyp3	LOC100192917	4	Protein of unknown function with a DUF3411 domain (NCBI).
HypE	LOC100275334	2	Predicted to be a member of the NADH-ubiquinone oxidoreductase complex I (NCBI).
HypF	LOC100283211	0	Similarity to chalcone isomerase (NCBI).
HypFD	LOC100282099	4	Put. PRA1-family protein. This protein family contains the glutamate transporter (EAAC1) interacting protein GTRAP3-18. Could regulate metabolite transport (Lin et al., 2001).
Mep1	LOC100383166	12	LrgB-like protein (Bräutigam et al., 2008).
Mep3	LOC100276525	4	Protein of unknown function with DUF3411 domain (Bräutigam et al., 2008; NCBI).
UP-a	LOC100285818	1	Proline-rich protein with similarity to members of the alpha-amylase inhibitors (AAI), lipid transfer (LT), and seed storage (SS) protein family (Kader, 1996, 1997; NCBI).
UP-d	LOC100192831	4	Belongs to the uncharacterized protein family, UPF0114 (NCBI).
UP-f	LOC100277914	1	Contains a MAEBL domain. MAEBL proteins were identified in *Plasmodium yoelii* and *P. falciparum* as type I transmembrane proteins with erythrocyte binding activity (Singh et al., 2004). Their function in plants is unknown.
PIC/TIC	LOC100273175	4	Contains a DUF3611. Similarity to the *A.t*. TIC21. Is predicted to be involved in copper homeostasis and protein import into chloroplasts.
PEPC	LOC100191762	0	Phosphoenolpyruvate carboxylase; initial carbon assimilation in the mesophyll cells of maize yielding oxaloacetate (Bräutigam et al., 2008).
NADP-ME	NP_001105313	2	NADP-Malic enzyme; reduction of malate in the BS chloroplast yielding CO_2_, NADPH and pyruvate (Bräutigam et al., 2008).

The abbreviations for proteins are identical to those used in other tables and in the figures. Number of transmembrane regions was determined as described in Section “Materials and Methods.”

**Table 2:** **Summary table showing membrane-associated proteins and their predicted or tested localization and bundle sheath (BS) or mesophyll (M) association**.

Protein name	Localization (GFP/YFP or pred.)	No. Of TM regions (Octopus)	Spectral count ratio (BS/M)	BS/M ratio determined by RT-PCR
5TM	YFP	8	0.79	0.58 ± 0.11
ER-AP	GFP	3	0.70	0.65 ± 0.02
Hyp2	unknown	11	0.57	0.70 ± 0.27
Hyp3	Cp-pred	4	BS	0.87 ± 0.25
HypE	Cp-pred	2	0.80	0.78 ± 0.14
HypF	Cp-pred	0	0.57	0.65 ± 0.23
HypFD	Cp-pred	4	0.31	0.28 ± 0.15
Mep1	Cp-GFP	12	1.09	1.12 ± 0.56
Mep3	Cp-YFP	4	0.69	3.00 ± 1.56
UP-a	Cp-YFP	1	1.41	0.66 ± 0.11
UP-d	Cp-YFP	4	1.05	0.40 ± 0.05
UP-f	CP-pred	1	3.09	0.84 ± 0.09
PIC/TIC	Cp-YFP	4	1.20	0.99 ± 0.16
PEPC	Cyt	0	0.71	0.30 ± 0.20
NADP-ME	Cp	2	5.32	4.44 ± 1.57

**Figure 4:**
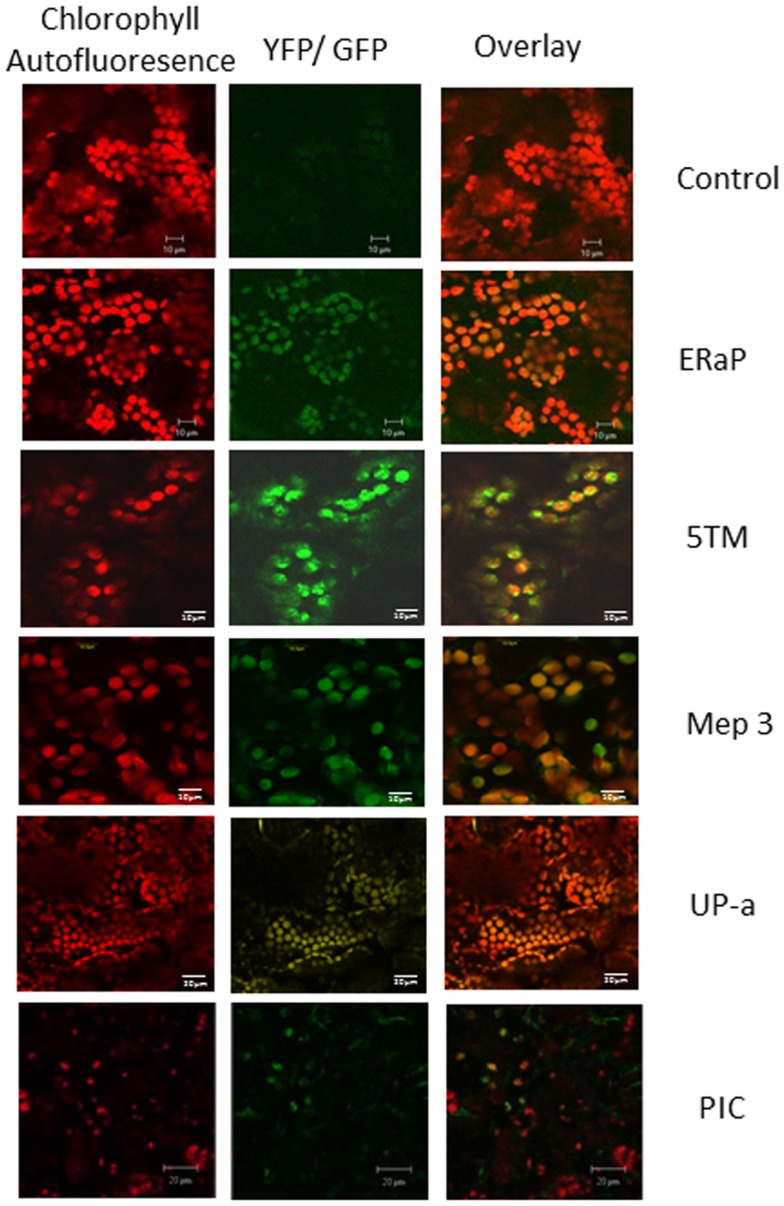
**Localization of chloroplast envelope proteins with C-terminal GFP or YFP tags that were transiently expressed in *Nicotiana tabacum***. The first column shows chlorophyll autofluorescence, the second column GFP or YFP fluorescence, column three shows the overlay. Protein names (as used in Table 1) are indicated on the right of each row.

*Localization prediction was based on computer prediction or on actual GFP/YFP labeling (Figure 4). The spectral count ratio was taken from the proteomics data (Table S2 in Supplementary Material; Figure 2); gene expression ratios were obtained by RT-PCR (Figure 3). The abbreviations for proteins are identical to those used in both supplementary tables. Spectral count ratios are based on the average of three experiments; RT-PCR-based ratios are mean and standard error of three experiments*.

The spectral count ratio had suggested that equal amounts of 5TM, Mep1, HypE, UP-a, UP-d, ER-AP, Mep3, and PIC/TIC are present in mesophyll and BS envelopes. Hyp2, HypF, Hyp FD are more abundant in the mesophyll cells, while Hyp3 and UP-f are slightly more abundant in the BS envelope. In most cases and in the two controls, the difference in relative amount of protein between BS and mesophyll chloroplast envelopes is closely correlated with the expression of the respective genes (within the margin of error).

Mep1 is a predicted LrgB-like protein. It is predicted to have 12 membrane-spanning regions. Mep1 (mesophyll envelope protein1) is enriched in the chloroplasts of the C4 plant maize relative to the C3 plant pea, but its gene expression is evenly distributed between BS and M cells in corn (Bräutigam et al., 2008). Based on our spectral count ratio, this is also true for the protein level (see Table 2).

ER-AP showed marginal differences in abundance between BS and M cells that were shown to be not significant using a student’s *t*-test (see Table S3 and S4 in Supplementary Material). This is likely a consequence of the fluctuation in spectral ion counts between different samples and could be a due to poor ionization of the tryptic fragments or dissociation from the membrane. However, since ER-AP is predicted to be ER-associated and it has been shown that the ER is in contact with chloroplasts, the large variability in ER-AP protein abundance may be a result of different degrees of chloroplast-ER interactions (Andersson et al., 2007).

The direct relationship between gene expression and protein abundance, however, is not true for all proteins: the two proteins, which were assigned to the BS, based on spectral ion counts, show equal gene expression levels in both M and BS cells. In the case of Hyp3, this may be due to a generally small amount of the protein within our samples. On the other hand, while Mep3 protein is present in equal quantities in BS and M cell, gene expression appears to be increased in BS cells. Given that several of these proteins had been detected in chloroplasts by other groups, contamination seems unlikely. On the other hand, it has been shown that protein abundance and mRNA levels do not necessarily correlate, especially in plastids (Li et al., 2010). These authors calculated BS versus M localization based on RNA-Seq data and compared those to a proteomics data set (Friso et al., 2010). They assigned ER-AP, UP-d, and PEPC to the mesophyll, while Mep1, Mep3, and NADP-ME were allocated to the BS cells. Yet, they found correlation between their data and protein abundances in some but not all cases (for example BS/M ratio for Mep1: 0.8 based on proteomics, 2.0 based on RNA-Seq). Possible explanations could be that either mRNA or proteins are more stable in the BS or that the protein is not imported into the mesophyll envelope. This would suggest further mechanisms controlling the turnover and incorporation of chloroplast envelope proteins.

### Confirmation of chloroplast localization of select chloroplast envelope proteins

To confirm the chloroplast localization of the above proteins, we cloned the respective genes with a carboxy-terminal GFP or YFP tag using the Gateway cloning system and transiently expressed them in *Nicotiana tabacum* (Figure 4). ER-AP, Mep 3, UP-a, Hyp g, and Hyp d show an even co-localization with the chloroplast, suggesting they are present in the plastid. PIC and 5TM show a spotted pattern that is clearly associated with the chloroplast but appears to be on the surface of the plastid. The pattern is similar to the one observed for multiple inner envelope proteins (Breuers et al., 2012).

### Distribution of gene expression throughout the plant and during transition to light

**Figure 5:**
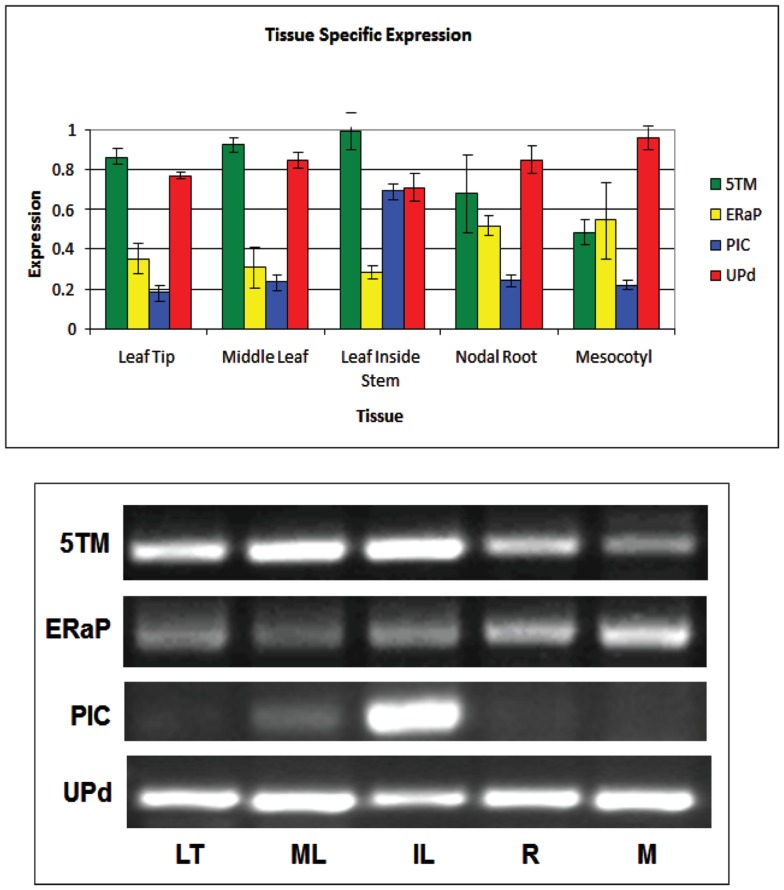
**Quantification of semiquantitative RT-PCR showing tissue-specific expression of genes encoding the 5TM protein (green bars), ER-AP (yellow bars), PIC-like protein (blue bars), and UP-d (red bars)**. Representative gel pictures are shown in the lower half. Band intensities of three to five biological replicates were quantified and displayed in the bar graph.

**Figure 6:**
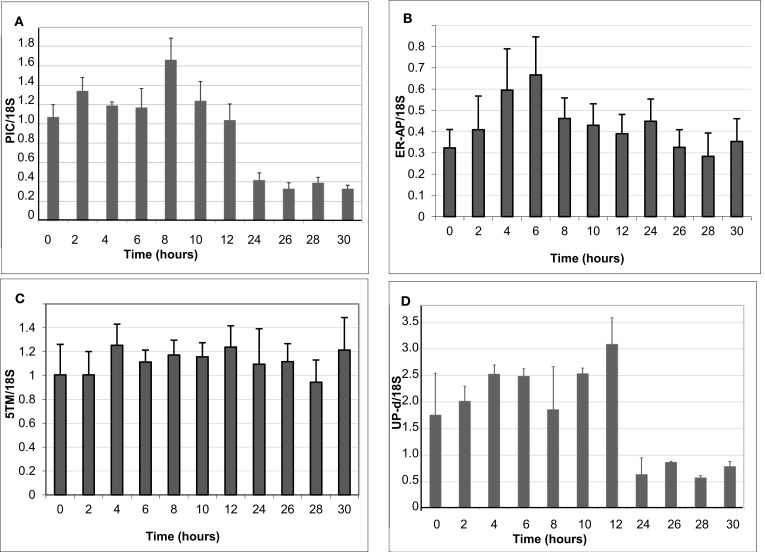
**Change in the expression of genes encoding the PIC-like protein (A), ER-AP (B), 5TM protein (C), and UP-d (D)**. Values were obtained from five to six biological replicates.

To better understand if and how 5TM, ER-AP, UP-d, and PIC could be associated with chloroplast development and/or function in relation to C4 photosynthesis, we studied their gene expression throughout the plant as well as at different times during leaf development and normalized them based on 18S expression levels (Figure 5). Leaf samples were taken at the tip (fully developed and expanded; source tissue; part 1 and 2 according to Pick et al., 2011), center (fully developed; expanding; source tissue; part 5 according to Pick et al., 2011), and at the base inside the sheath (etiolated; expanding; sink tissue). To investigate a possible role in chloroplast development, expression was also monitored during the transition of 5-day-old dark-grown seedling into light (Figure 6). Primary leaves in these seedlings started to turn green within 3–5 h, a process that was completed by 24 h.

The gene for 5TM shows high expression in all parts of the leaf but is also expressed in the root and the mesocotyl in 6-week-old plants. Similarly, if 6-day-old etiolated corn seedlings are moved to light, 5TM gene expression in the leaves is constitutively high over a 30-h time period after transition to light (Figures 5 and 6C). This suggests its function likely is not related to photosynthesis. Similarly, there is no significant change of Up-d expression during transition to light (Figure 6D).

PIC expression is present mostly in parts of the leaf that were located within the sheath and not exposed to light and to a much smaller extent in the green part of the leaf (Figure 5). During transition to light, it shows a transient 60% increase for the first 8 h before being gradually reduced to 50% of the expression at the start of the exposure (Figure 6A). This could indicate a role either in chloroplast development or in processes that are present in the dark and cease after transition to light.

*ER-AP* gene expression appears to be slightly higher in roots and mesocotyl than in leaves (Figure 5). When moved to light, however, ER-AP expression more than doubled within 4–6 h, followed by a return to dark-grown levels within the next 6 h (Figure 6B). This increase was statistically significant (*p* < 0.05 for the 4, 8, and 10-h time points and *p* < 0.1 for the 6-h time point). This suggests that while the gene product may be necessary for general cellular functions, it may also be relevant for the light-dependent transition from proplastids to chloroplasts.

## Conclusion

A large number of chloroplast proteins and putative metabolite transporters have already been identified through proteomics experiments. In addition, genome databases have increased the number of candidates. We have shown here that despite this large candidate pool, further fractionation can still lead to the discovery of novel proteins. To make these protein lists meaningful, it is now necessary to characterize bioinformatics predictions. We have confirmed the chloroplast association of seven of our identified chloroplast envelope proteins. Based on gene expression studies throughout the plant and during transition to light, we conclude that the 5TM and the UP-d protein may not relevant for chloroplast development or C4 metabolite transport, but that ER-AP and PIC constitute good candidates for further study.

## Conflict of Interest Statement

The authors declare that the research was conducted in the absence of any commercial or financial relationships that could be construed as a potential conflict of interest.

## Supplementary Material

The Supplementary Material for this article can be found online at http://www.frontiersin.org/Plant_Proteomics/10.3389/fpls.2013.00065/abstract

Supplementary Table S1 **Primers used for RT-PCR experiments**.

Supplementary Table S2 **Proteins identified in bundle sheath and mesophyll envelopes**.

Supplementary Table S3 **Raw data and calculation of BS/M ratio based on spectral ion count**.

Supplementary Table S4 **Proteins with differential abundance that are depicted in Figure 2**.

## Footnotes

^1^www.proteomesoftware.com

^2^ftp://occams.dfci.harvard.edu/pub/bio/tgi/data/Zea_mays

^3^http://ppdb.tc.cornell.edu

^4^www.psort.org

## References

[B1] Altschul S. F. Madden T. L. Schaffer A. A. Zhang J. H. Zhang Z. Miller W. (1997). Gapped BLAST and PSI-BLAST: a new generation of protein database search programs. Nucleic Acids Res. 25, 3389–3402 10.1093/nar/25.17.3389 9254694 PMC146917

[B2] Andersson M. X. Goksör M. Sandelius A. S. (2007). Optical manipulation reveals strong attracting forces at membrane contact sites between endoplasmic reticulum and chloroplasts. J. Biol. Chem. 282, 1170–1174 10.1074/jbc.M706129200 17077082

[B3] Bassham J. Benson A. Calvin M. (1950). The path of carbon in photosynthesis. J. Biol. Chem. 185, 781–787 14774424

[B4] Bolter B. Soll J. Hill K. Hemmler R. Wagner R. (1999). A rectifying ATP regulated solute channel in the chloroplastic outer envelope from pea. EMBO J. 18, 5505–5516 10.1093/emboj/18.20.5505 10523295 PMC1171619

[B5] Bräutigam A. Hoffmann-Benning S. Weber A. P. M. (2008). Comparative proteomics of chloroplast envelopes from C3 and C4 plants reveals specific adaptations of the plastid envelope to C4 photosynthesis and candidate proteins required for maintaining C4 metabolite fluxes. Plant Physiol. 148, 568–579 10.1104/pp.108.121012 18599648 PMC2528119

[B6] Breuers F. K. Bräutigam A. Geimer S. Welzel U. Y. Stefano G. Renna L. (2012). Dynamic remodeling of the plastid envelope membranes – a tool for chloroplast envelope in vivo localizations. Front. Plant Sci 3:7 10.3389/fpls.2012.00007 22645566 PMC3355811

[B7] Breuers F. K. Bräutigam A. Weber A. P. (2011). The plastid outer envelope – a highly dynamic interface between plastid and cytoplasm. Front. Plant Sci. 2:97 10.3389/fpls.2011.00097 22629266 PMC3355566

[B8] Cline K. Andrews J. Mersey B. Newcomb E. H. Kenneth K. (1981). Separation and characterization of inner and outer envelope membranes of pea chloroplasts. Proc. Natl. Acad. Sci. U.S.A. 78, 3595–3599 10.1073/pnas.78.6.3595 16593034 PMC319617

[B9] Earley K. W. Haag J. R. Pontes O. Opper K. Juehne T. Song K. (2006). Gateway-compatible vectors for plant functional genomics and proteomics. Plant J. 45, 616–629 10.1111/j.1365-313X.2005.02617.x 16441352

[B10] Edwards G. E. Furbank R. T. Hatch M. D. Osmond C. B. (2001). What does it take to be C4? Lessons from the evolution of C4 photosynthesis. Plant Physiol. 125, 46–49 10.1104/pp.125.1.46 11154293 PMC1539322

[B11] Emanuelsson O. Nielsen H. Brunak S. von Heijne G. (2000). Predicting subcellular localization of proteins based on their N-terminal amino acid sequence. J. Mol. Biol. 300, 1004–1016 10.1006/jmbi.2000.3903 10891285

[B12] Emanuelsson O. Nielsen H. von Heijne G. (1999). ChloroP, a neural network-based method for predicting chloroplast transit peptides and their cleavage sites. Protein Sci. 8, 978–984 10.1110/ps.8.5.978 10338008 PMC2144330

[B13] Ferro M. Brugière S. Salvi D. Seigneurin-Berny D. Court M. Moyet L. (2010). AT_CHLORO, a comprehensive chloroplast proteome database with subplastidial localization and curated information on envelope proteins. Mol. Cell. Proteomics 9, 1063–1084 10.1074/mcp.M900325-MCP200 20061580 PMC2877971

[B14] Ferro M. Salvi D. Brugiere S. Miras S. Kowalski S. Louwagie M. (2003). Proteomics of the chloroplast envelope membranes from Arabidopsis thaliana. Mol. Cell. Proteomics 2, 325–345 12766230 10.1074/mcp.M300030-MCP200

[B15] Fischer K. (2011). The import and export business in plastids: transport processes across the inner envelope membrane. Plant Physiol. 155, 1511–1519 10.1104/pp.110.170241 21263040 PMC3091126

[B16] Friso G. Giacomelli L. Ytterberg A. J. Peltier J. B. Rudella A. Sun Q. (2004). In-depth analysis of the thylakoid membrane proteome of Arabidopsis thaliana chloroplasts: new proteins, new functions, and a plastid proteome database. Plant Cell 16, 478–499 10.1105/tpc.017814 14729914 PMC341918

[B17] Friso G. Majeran W. Huang M. Sun Q. van Wijk K. J. (2010). Reconstruction of metabolic pathways, protein expression, and homeostasis machineries across maize bundle sheath and mesophyll chloroplasts: large-scale quantitative proteomics using the first maize genome assembly. Plant Physiol. 152, 1219–1250 10.1104/pp.109.152694 20089766 PMC2832236

[B18] Froehlich J. E. Wilkerson C. G. Ray W. K. McAndrew R. S. Osteryoung K. W. Gage D. A. (2003). Proteomic study of the Arabidopsis thaliana chloroplastic envelope membrane utilizing alternatives to traditional two-dimensional electrophoresis. J. Proteome Res. 2, 413–425 10.1021/pr034025j 12938931

[B19] Goetze T. A. Philippar K. Ilkavets I. Soll J. Wagner R. (2006). OEP37 is a new member of the chloroplast outer membrane ion channels. J. Biol. Chem. 281, 17989–17998 10.1074/jbc.M600700200 16624824

[B20] Hatch M. D. (1987). C-4 photosynthesis: a unique blend of modified biochemistry, anatomy and ultrastructure. Biochim. Biophys. Acta 895, 81–106 10.1016/S0304-4173(87)80009-5

[B21] Heber U. Walker D. (1992). Concerning a dual function of coupled cyclic electron transport in leaves. Plant Physiol. 100, 1621–1626 10.1104/pp.100.4.1621 16653176 PMC1075843

[B22] Horton P. Park K.-J. Obayashi T. Nakai K. (2006). “Protein subcellular localization prediction with WoLF PSORT,” in Proceedings of Asian Pacific Bioinformatics Conference 2006, Taipei

[B23] Jarvis P. Soll J. (2002). Toc, tic, and chloroplast protein import. Biochim. Biophys. Acta 1590, 177–189 10.1016/S0167-4889(02)00176-3 12180471

[B24] Kader J.-C. (1996). Lipid transfer proteins in plants. Annu. Rev. Plant Physiol. Plant Mol. Biol. 47, 627–654 10.1146/annurev.arplant.47.1.627 15012303

[B25] Kader J.-C. (1997). Lipid transfer proteins: a puzzling family of plant proteins. Trends Plant Sci. 2, 66–70 10.5363/tits.2.11_66

[B26] Kanai R. Edwards G. E. (1973). Separation of mesophyll protoplasts and bundle sheath cells from maize leaves for photosynthetic studies. Plant Physiol. 51, 1133–1137 10.1104/pp.51.6.1133 16658479 PMC366418

[B27] Kato Y. Miura E. Ido K. Ifuku K. Sakamoto W. (2009). The variegated mutants lacking chloroplastic FtsHs are defective in D1 degradation and accumulate reactive oxygen species. Plant Physiol. 151, 1790–1801 10.1104/pp.109.146589 19767385 PMC2785964

[B28] Keller A. Nesvizhskii A. I. Kolker E. Aebersold R. (2002). Empirical statistical model to estimate the accuracy of peptide identifications made by MS/MS and database search. Anal. Chem. 74, 5383–5392 10.1021/ac025747h 12403597

[B29] Kinoshita H. Nagasaki J. Yoshikawa N. Yamamoto A. Takito S. Kawasaki M. (2011). The chloroplastic 2-oxoglutarate/malate transporter has dual function as the malate valve and in carbon/nitrogen metabolism. Plant J. 65, 15–26 10.1111/j.1365-313X.2010.04397.x 21175886

[B30] Kleffmann T. Hirsch-Hoffmann M. Gruissem W. Baginsky S. (2006). Plprot: a comprehensive proteome database for different plastid types. Plant Cell Physiol. 47, 432–436 10.1093/pcp/pcj005 16418230

[B31] Kleffmann T. Russenberger D. Von Zychlinski A. Christopher W. Sjolander K. Gruissem W. (2004). The Arabidopsis thaliana chloroplast proteome reveals pathway abundance and novel protein functions. Curr. Biol. 14, 354–362 10.1016/j.cub.2004.02.039 15028209

[B32] Kriechbaumer V. Nabok A. Mustafa M. K. Al-Ammar R. Tsargorodskaya A. Smith D. P. (2012). Analysis of protein interactions at native chloroplast membranes by ellipsometry. PLoS ONE 7:e34455 10.1371/journal.pone.0034455 22479632 PMC3315527

[B33] Leegood R. C. Edwards G. E. (1996). Photosynthesis and the Environment, Vol. 5 Dordrecht: Kluwer Academic Publishers

[B34] Li P. Ponnala L. Gandotra N. Wang L. Si Y. Tausta S. (2010). The developmental dynamics of the maize leaf transcriptome. Nat. Genet. 42, 1060–1067 10.1038/ng.680 21037569

[B35] Lin C. I. Orlov I. Ruggiero A. M. Dykes-Hoberg M. Lee A. Jackson M. (2001). Modulation of the neuronal glutamate transporter EAAC1 by the interacting protein GTRAP3-18. Nature 410, 84–88 10.1038/35065084 11242046

[B36] Linka N. Weber A. P. M. (2010). Intracellular metabolite transporters in plants. Mol. Plant 3, 1–33 10.1093/mp/ssp108 20038549

[B37] Liu H. Sadygov R. G. Yates J. R. (2004). A model for random sampling and estimation of relative protein abundance in shotgun proteomics. Anal. Chem. 76, 4193–4201 10.1021/ac049573a 15253663

[B38] Lu P. Vogel C. Wang R. Yao X. Marcotte E. M. (2007). Absolute protein. Nat. Biotechnol. 25, 117–124 10.1038/nbt1207-1403 17187058

[B39] Lundquist P. K. Poliakov A. Bhuiyan N. H. Zybailov B. Sun Q. van Wijk K. J. (2012). The functional network of the Arabidopsis plastoglobule proteome based on quantitative proteomics and genome-wide coexpression analysis. Plant Physiol. 158, 1172–1192 10.1104/pp.111.193144 22274653 PMC3291262

[B40] Majeran W. Cai Y. Sun Q. vanWijk K. J. (2005). Functional differentiation of bundle sheath and mesophyll maize chloroplasts determined by comparative proteomics. Plant Cell 17, 3111–3140 10.1105/tpc.105.035519 16243905 PMC1276033

[B41] Majeran W. Friso G. Asakura Y. Qu X. Huang M. Ponnala L. (2012). Nucleoid-enriched proteomes in developing plastids and chloroplasts from maize leaves: a new conceptual framework for nucleoid functions. Plant Physiol. 158, 156–189 10.1104/pp.111.188474 22065420 PMC3252073

[B42] Majeran W. van Wijk K. J. (2009). Cell-type-specific differentiation of chloroplasts in C4 plants. Trends Plant Sci. 14, 100–109 10.1016/j.tplants.2008.11.006 19162526

[B43] Majeran W. Zybailov B. Ytterberg A. J. Dunsmore J. Sun Q. van Wijk K. J. (2008). Consequences of C4 differentiation for chloroplast membrane proteomes in maize mesophyll and bundle sheath cells. Mol. Cell Proteomics 79, 1609–1638 18453340 10.1074/mcp.M800016-MCP200 PMC2556027

[B44] Marchler-Bauer A. Lu S. Anderson J. B. Chitsaz F. Derbyshire M. K. DeWeese-Scott C. (2011). CDD: a Conserved Domain Database for the functional annotation of proteins. Nucleic Acids Res. 39D, 225–229 10.1093/nar/gkq1189 PMC3013737 21109532

[B45] Matsuoka M. Nomura M. Agarie S. Miyao-Tokutomi M. Ku M. S. B. (1998). Evolution of C4 photosynthetic genes and overexpression of maize C4 genes in rice. J. Plant Res. 111, 333–337 10.1007/BF02512193

[B46] Meierhoff K. Westhoff P. (1993). Differential biogenesis of photosystem II in mesophyll and bundle-sheath cells of monocotyledonous NADP-malic enzyme-type C4 plants: the non-stoichiometric abundance of the subunits of photosystem II in the bundle-sheath chloroplasts and the translational activity of the plastome-encoded genes. Planta 191, 23–33 10.1007/BF00240892

[B47] Nziengui H. Bouhidel K. Pillon D. Der C. Marty F. Schoefs B. (2007). Reticulon-like proteins in *Arabidopsis thaliana*: structural organization and ER localization. FEBS Lett. 581, 3356–3362 10.1016/j.febslet.2007.06.032 17604024

[B48] Peltier J. B. Emanuelsson O. Kalume D. E. Ytterberg J. Friso G. Rudella A. (2002). Central functions of the lumenal and peripheral thylakoid proteome of Arabidopsis determined by experimentation and genome-wide prediction. Plant Cell 14, 211–236 10.1105/tpc.010304 11826309 PMC150561

[B49] Peltier J. B. Ripoll D. R. Friso G. Rudella A. Cai Y. Ytterberg J. (2004). Clp protease complexes from photosynthetic and non-photosynthetic plastids andmitochondria of plants, their predicted three-dimensional structures, and functional implications. J. Biol. Chem. 279, 4768–4781 10.1074/jbc.M406763200 14593120

[B50] Pick T. R. Bräutigam A. Schlüter U. Denton A. K. Colmsee C. Scholz U. (2011). Systems analysis of a maize leaf developmental gradient redefines the current C4 model and provides candidates for regulation. Plant Cell 12, 4208–4220 22186372 10.1105/tpc.111.090324 PMC3269860

[B51] Pohlmeyer K. Soll J. Grimm R. Hill K. Wagner R. (1998). A high-conductance solute channel in the chloroplastic outer envelope from pea. Plant Cell 10, 1207–1216 10.2307/3870722 9668138 PMC144050

[B52] Pohlmeyer K. Soll J. Steinkamp T. Hinnah S. Wagner R. (1997). Isolation and characterization of an amino acid-selective channel protein present in the chloroplastic outer envelope membrane. Proc. Natl. Acad. Sci. U.S.A. 94, 9504–9509 10.1073/pnas.94.17.9504 9256512 PMC23240

[B53] Rochaix J.-D. (2011). Regulation of photosynthetic electron transport. Biochim. Biophys. Acta 1807, 878–886 10.1016/j.bbabio.2011.05.009 21605544

[B54] Roston R. Gao J. Xu C. Benning C. (2011). Arabidopsis chloroplast lipid transport protein TGD2 disrupts membranes and is part of a large complex. Plant J. 66, 759–769 10.1111/j.1365-313X.2011.04536.x 21309871

[B55] Sage R. F. (2004). The evolution of C-4 photosynthesis. New Phytol. 161, 341–370 10.1111/j.1469-8137.2004.00974.x 33873498

[B56] Schleiff E. Eichacker L. A. Eckart K. Becker T. Mirus O. Stahl T. (2003). Prediction of the plant beta-barrel proteome: a case study of the chloroplast outer envelope. Protein Sci. 12, 748–759 10.1110/ps.0237503 12649433 PMC2323836

[B57] Shevchenko A. Wilm M. Vorm O. Mann M. (1996). Mass spectrometric sequencing of proteins from silver-stained polyacrylamide gels. Anal. Chem. 68, 850–858 10.1021/ac950914h 8779443

[B58] Shikani T. (2007). Cyclic electron transport around photosystem I: genetic approaches. Annu. Rev. Plant Biol. 58, 199–217 10.1146/annurev.arplant.58.091406.110525 17201689

[B59] Siddique M. A. Grossmann J. Gruissem W. Baginsky S. (2006). Proteome analysis of bell pepper (Capsicum annuum L.) chromoplasts. Plant Cell Physiol. 47, 1663–1673 10.1093/pcp/pcl033 17098784

[B60] Singh N. Preiser P. Rénia L. Balu B. Barnwell J. Blair P. (2004). Conservation and developmental control of alternative splicing in maebl among malaria parasites. J. Mol. Biol. 343, 589–585 10.1016/j.jmb.2004.08.047 15465047

[B61] Sirpiö S. Allahverdiyeva Y. Suorsa M. Paakkarinen V. Vainonen J. Battchikova N. (2007). TLP18.3, a novel thylakoid lumen protein regulating photosystem II repair cycle. Biochem. J. 406, 415–425 10.1042/BJ20070460 17576201 PMC2049043

[B62] Slack C. R. Hatch M. D. Goodchild J. (1969). Distribution of enzymes in mesophyll and parenchyma-sheath chloroplasts of maize leaves in relation to the C4-dicarboxylic acid pathway of photosynthesis. Biochem. J. 114 489–498 4309527 10.1042/bj1140489 PMC1184920

[B63] Taiz L. Zeiger E. (eds). (2006). Plant Physiology, 4th Edn. Sunderland: Sinauer Associates Inc

[B64] Taniguchi Y. Nagasaki J. Kawasaki M. Miyake H. Sugiyama T. Taniguchi M. (2004). Differentiation of dicarboxylate transporters in mesophyll and bundle sheath chloroplasts of maize. Plant Cell Physiol. 45, 187–200 10.1093/pcp/pch022 14988489

[B65] Tyra H. M. Linka M. Weber A. P. Bhattacharya D. (2007). Host origin of plastid solute transporters in the first photosynthetic eukaryotes. Genome Biol. 8, R212 10.1186/gb-2007-8-5-212 17919328 PMC2246286

[B66] Viklund H. Elofsson A. (2008). Improving topology prediction by two-track ANN-based preference scores and an extended topological grammar. Bioinformatics 24, 1662–1668 10.1093/bioinformatics/btn550 18474507

[B67] von Zychlinski A. Kleffmann T. Krishnamurthy N. Baginsky S. Gruissem W. (2005). Proteome analysis of the rice etioplast: metabolic and regulatory networks and novel protein functions. Mol. Cell. Proteomics 4, 1072–1084 10.1074/mcp.M500018-MCP200 15901827

[B68] Weber A. P. M. (2004). Solute transporters as connecting elements between cytosol and plastid stroma. Curr.Opin. Plant Biol. 7, 247–253 10.1016/j.pbi.2004.03.008 15134744

[B69] Weber A. P. M. (2010). Intracellular metabolite transporters in plants. Mol Plant 3, 1–33 10.1093/mp/ssp111 20038549

[B70] Weber A. P. M. Fischer K. (2007). Making the connections: the crucial role of metabolite transporters at the interface between chloroplast and cytosol. FEBS Lett. 581, 2215–2222 10.1016/j.febslet.2007.02.010 17316618

[B71] Weber A. P. M. Schwacke R. Flügge U. I. (2005). Solute transporters of the plastid envelope membrane. Annu. Rev. Plant Biol. 56, 133–164 10.1146/annurev.arplant.56.032604.144228 15862092

[B72] Winter D. Vinegar B. Nahal H. Ammar R. Wilson G. V. Provart N. J. (2007). An “electronic fluorescent pictograph” browser for exploring and analyzing large-scale biological data sets. PLoS ONE 2:e718 10.1371/journal.pone.0000718 17684564 PMC1934936

[B73] Xiao W. Sheen J. Jang J. C. (2000). The role of hexokinase in plant sugar signal transduction and growth and development. Plant Mol. Biol. 44, 451–461 10.1023/A:1026501430422 11197321

[B74] Zybailov B. Coleman M. K. Florens L. Washburn M. P. (2005). Correlation of relative abundance ratios derived from peptide ion chromatograms and spectrum counting for quantitative proteomic analysis using stable isotope labeling. Anal. Chem. 77, 6218–6224 10.1021/ac050846r 16194081

