# Supplementary material for: Comparative Proteomics of Chloroplasts Envelopes from Bundle Sheath and Mesophyll Chloroplasts Reveals Novel Membrane Proteins with a Possible Role in C4-Related Metabolite Fluxes and Development
Source: Front Plant Sci. 2013 Mar 28;4:65. doi: 10.3389/fpls.2013.00065 (PMC3610082; doi:10.3389/fpls.2013.00065)
Supplement: Supplementary Table S1 — Primers used for RT-PCR experiments. [file 42937_Hoffmann-Benning_DataSheet1.DOCX]

Supplementary table 1:

Primers used for RT-PCR: primers were designed based on sequences identified through T C and TA numbers if given.

|  | |  |  |
| --- | --- | --- | --- |
| Gene Name | LOC number | TA/TC Number | PCR Primers |
| Mep1 | LOC100383166 | TC319539_6 | CACCTCGCCTGCTAGCCTCCTCC |
|  |  |  | CAGTGCTGCTGTTGGTCGGACC |
| Mep3 | LOC100276525 | TC354958_5 | CCTCCCTCACAAAAACCTACCACC |
|  |  |  | GCTTCCGGTGATGTCGCTTCC |
| 5TM | LOC100283913 | TC351680 | CAA AGA CCG GAA CCT AGA G |
|  |  |  | CTG TGT TTC TTG CAG GTG |
| Hyp2 | LOC100285177 | TC321961_5 | GGC CAA CAA GGC CGA GGA GA |
|  |  |  | TCT TTA CTC CCG GAC AGG CCA GA |
| Hyp3 | LOC100192917 | TA180753_4577 | CCA TCC GCA TCG CTG CCC TG |
|  |  |  | TCG AGC GTG ACA GCC CCC AA |
| Hyp E | LOC100275334 | TC337274_4 | GCACGACGGCGACCAAATTCTGC |
|  |  |  | GTTGCCGACCACCTTGGTGAAGG |
| Hyp F | LOC100283211 | TA149497_4577 | GCC ATC CCA GGC CCC CAA C |
|  |  |  | CGC ATA CAC GCC GAA TGC G |
| Hyp FD | LOC100282099 | TC317888_5 | CCACTTCGCCATGAACTATGCCA |
|  |  |  | CTGGAGGAACTCGGAAACAGGGA |
| PIC | LOC100273175 | TC347419_6 | GTCAGGAGGACCCCAGCCCTCC |
|  |  |  | GAAGACGCAGATGAAGGCCACGA |
| UP-a | LOC100285818 | TC340018 | GCG GCG AAC GCT ATG GGC AA |
|  |  |  | CCC CAG CAT CAG GTG AGT CC |
| Up-d | LOC100192831 |  | GCTACTCGATCTGCATCTTC |
|  |  |  | CCAACCAAAGCAATAGGC |
| UP-f | LOC100277914 | TC342873 | ACT CGA CCC GAA ATG GAC ACT TGA |
|  |  |  | AAG ATG GGA AGC GCC ACG CC |
| ER -AP | LOC100283096 | TC348969_5 | CTG CCT CTC CGG CGC CAT TG |
|  |  |  | ATG TTG CCC ATC AGG TCC GC |
| Malic enzyme | NP_001105313 | TA105625_4577 | CCA CAC TGC CAA CTC GCT CCG |
|  |  |  | GAC CGC GTC CAC CCG CCT G |
| 18S | AF168884 |  | TCAACTTTCGATGGTAGGATAGTG |
|  |  |  | CCGTGTCAGGATTGGGTAATTT |
| PEPC | LOC100191762 |  | TTCCGTGTCTAGCTTCCCG |
|  |  |  | GGCGAACCCACCTTTCTTG |
| Rubisco SSU | LOC100279574 |  | GGCCTACGGCAACAAGAAG |
|  |  |  | GGGCTTGTAGGCGATGAAG |
|  |  |  |  |
